# The Spontaneous Disappearance of Malignant Growths

**Published:** 1908-03-14

**Authors:** 


### Points in Surgery.

### THE SPONTANEOUS DISAPPEARANCE OF MALIGNANT GROWTHS.

The disappearance of a tumour under medical treatment, professional or quack, is an event of no very great rarity, though it does not always follow that any causal relation has existed. Further, the disappearance of tumours diagnosed as malignant has, though rare, been many times reported and discussed. More exceptionally still, the malignant nature of such growths has been established by microscopical examination, and yet they have spontaneously resolved.

These facts, familiar enough to the followers of cancer research, should be taken into consideration by any medical man who undertakes to deliver a prognosis on a case of malignant disease. Not for a moment do they bear on treatment, for instant and extensive surgical interference whenever possible is as yet our only remedy; but they do warn us against a too confident prognosis of evil, even in an apparently hopeless case where a microscopical report has confirmed the diagnosis. There is no need to discuss any case in which the diagnosis has not been thus established; amongst others reported are two remarkable cases, one of carcinoma and one of melanotic sarcoma, on which one may rely for proof of the danger of forecasting with certainty the result of a "hopeless" case.

The first is that of a patient shown by Mr. A. P. Gould at the Clinical Society in 1896, and again in 1899. This was a woman who, at the age of 33, was struck on the left breast with an umbrella in 1885. Three years after that she noticed a lump in her breast, and two years later still she came under the care of a surgeon, who amputated the organ. A scirrhous cancer was found at the microscopical examination. In 1892 the same surgeon removed glands from her axilla, and in 1894 recurrences about the scar and a tumour in the right breast were dealt with.

It was in the following year that she came under the care of Mr. Gould, who admitted her to hospital. On the left side of the chest were numerous nodules involving the skin and subcutaneous tissues, and in the left axilla several hard enlarged glands. Under the scar in the right breast was a tumour, and the axilla on that side also contained palpable glands; moreover, there were enlarged glands above both clavicles. The patient suffered from paroxysmal dyspnœa with abundant expectoration. When pain in the left thigh with enlargement of the bone and shortening, added to orthopnœa, cyanosis and hæmoptyses developed in 1896, it would have been difficult to imagine a case in which more justification for a fatal prognosis existed. Yet without treatment the patient recovered completely, but for a limp due to an inch and a half of shortening in the affected thigh.

The other case is perhaps more extraordinary still, for the microscope was called in three separate times. The details were published in 1899 by Sir W. H. Bennett;* but they will bear repetition, for the woman was seen only two or three years ago, quite well in spite of alcoholic indulgence meanwhile. The patient, then 48 years of age, first noticed in 1894 the growth of a painless black patch under the skin of the sole of the left foot. Nearly two years later this ulcerated, and was completely excised; a melanotic sarcoma was reported after a microscopical examination. Four months later there was local recurrence, and a Syme's amputation was performed; melanotic sarcoma was again reported, but with a diminished amount of pigment. Within three weeks of the operation many nodular subcutaneous tumours appeared in the left thigh, and at the same time the inguinal glands became enlarged. One of the tumours was excised, and described after examination as sarcoma with very little pigment. The number of tumours had now reached sixty-two, not counting the glands; the patient was advised that nothing further could be done, and she was discharged, as it was supposed, to die. Some of the tumours ulcerated and discharged externally; others disappeared spontaneously, without treatment of any sort, and two years later Sir W. H. Bennett was able to report the complete disappearance of the growths and the almost complete disappearance of the inguinal glandular enlargement. When seen five and a half years later there was no trace of even the latter, but there were present on the left thigh four or five small vein varices such as were not seen elsewhere on the limbs.

These two cases, though sufficiently amazing, could no doubt be paralleled from the experience of many practitioners. The lesson they enforce is that rare cases of cancer, in which the diagnosis is beyond all doubt, and the condition of the patient seemingly desperate, do recover solely through the *vis medicatrix naturæ*. Admitting this, any theory of the origin and nature of cancer must explain those cases. On any parasitic theory the development of acquired immunity by a protective substance in the blood serum would naturally suggest itself. The corollary to this is that the serum of a patient who had thus recovered might possess specific virtues when transferred to the tissues of another sufferer from a similar kind of tumour. One hardly envies the lot of the man or woman who shall become known as the unconscious manufacturer of such a serum. There is nothing, in the light of our present knowledge of immunisation and serum therapy, wildly impossible in the conception; if cancer is due to a parasite at all homologous to the parasites of the various infective diseases, the serum of one who has, out of the resources of his own economy, conquered those parasites may contain for a certain length of time afterwards substances which would diminish the virulence of a similar infection in another patient. If someone could be found who had thus recovered, after being proved the subject of malignant disease, and public-spirited enough to surrender a quantity of his blood serum to a recognised research laboratory, the result would at least be of interest, and might be of the most far-reaching and intense importance.

* *Lancet*, Jan. 7, 1899.

